# Occurrences of *Sinolagomys* (Lagomorpha) from the Valley of Lakes (Mongolia)

**DOI:** 10.1007/s12549-016-0262-z

**Published:** 2017-01-25

**Authors:** Margarita Erbajeva, Bayarmaa Baatarjav, Gudrun Daxner-Höck, Lawrence J. Flynn

**Affiliations:** 1 Geological Institute, Siberian Branch, Russian Academy of Sciences, Sahianova Str., 6a, Ulan-Ude, 670047 Russia; 2 Institute of Palaeontology and Geology, Mongolian Academy of Sciences, Danzan Str., Post 46/650, Ulaanbaatar, Mongolia; 3 Rupertusstr. 16, 5201 Seekirchen, Austria; 4 Department of Human Evolutionary Biology, Harvard University, 11 Divinity Avenue, Cambridge, MA 02138 USA

**Keywords:** Ochotonidae, *Sinolagomys*, Dentition, Oligocene, Valley of Lakes, Mongolia

## Abstract

The genus *Sinolagomys* is an early representative of the family Ochotonidae, appearing first in the late early Oligocene of Central Asia. It is known in China from Shargaltein Tal, Taben Buluk, Ulan Tatal, and northern Junggaria, and a few specimens from Tatal Gol, Mongolia have been published. For most occurrences, the genus is not represented by many specimens. Extensive studies in the Valley of Lakes, Central Mongolia, have produced a large number of sinolagomyin fossils spanning at least 10 million years and belonging to five species: *Sinolagomys kansuensis*, *Sinolagomys major*, *Sinolagomys gracilis*, *Sinolagomys ulungurensis*, and *Sinolagomys badamae* sp. nov. Descriptions of these are given, as well as definition of the new species. Sinolagomyins flourished during the late Oligocene and early Miocene and came to occupy vast territories from China through Mongolia and Kazakhstan. The evolution of this ochotonid group is characterized by increasing taxonomic diversity and progressive development of rootless cheek teeth.

## Introduction

The Order Lagomorpha, originating in Central Asia likely during the Paleocene Epoch (Asher et al. 2005), was represented by a number of early stem group lagomorphs such as genera *Khaychina, Eomylus, Amar, Zagmys, Mimotona, Eurymylus, Gomphos*, placed variously in the higher taxa Anagalida and Mimotonidae. The earliest lagomorphs were small sized, first class herbivore consumers in early Cenozoic ecology. The Eocene climate in Asia changed, drying as it became continental due to the influence of the Antarctic ice accumulation (Wolfe 1971). Globally, the climate continued to remain warm and humid in some areas (Berggren and Prothero 1992), but tropical forest gradually declined, the most archaic stem group genera disappeared, and a number of lagomorphs died out at the transition from the Eocene to the Oligocene. Stem group Lagomorpha were replaced by the early genera of a more advanced grade (*Lushilagus*, *Shamolagus*, *Strenulagus*, *Gobiolagus*) and by the first paleolagid, the genus *Desmatolagus*.

The first investigation of lagomorphs from Central Asia, specifically the Taatsin Gol area, was conducted by the Central Asiatic Expedition of the American Museum of Natural History (AMNH) (Matthew and Granger 1923). Matthew and Granger were the first to describe new taxa of *Desmatolagus* from the Hsanda Gol Formation. However, they did not mention the existence of a different form (later known as *Sinolagomys*) among a thousand desmatolagin fossils. The genus *Sinolagomys* stored at the AMNH (New York) is represented by a single jaw fragment with p3-m1 under collection number “AMNH FM: 56648.”

Later, based on material collected by the joint Soviet–Mongolian Paleontological expeditions, Gureev (1960) described from the Tatal Gol locality ten different lagomorph taxa, mostly desmatolagins, among which three fragments of ochotonid lower jaw with variable structure of p3 were discovered. One of them was described as new, under the species name *Sinolagomys tatalgolicus*.

The genus *Sinolagomys* had been recognised for the first time by Bohlin (1937) from the Oligocene localities of Shargaltein Tal, in Northern China. Early studies of *Sinolagomys* led to the recognition of three species: *Sinolagomys kansuensis*, *Sinolagomys major*, and *Sinolagomys minor*, the latter renamed by Bohlin as *Sinolagomys gracilis* (Bohlin 1942).

On the basis of the Tatal Gol specimens and revision of other Chinese ochotonid species, Gureev was of the opinion that *Sinolagomys*, although unusual, belongs to family Ochotonidae (=Lagomyidae), at that time including surviving species of the Holarctic genus *Ochotona*. The genus *Sinolagomys* differs from typical *Ochotona* in the simple structure of the main diagnostic teeth (p3 and P4-M2) and by retaining reduced cheek teeth roots.

These data allowed Gureev to refer *Sinolagomys* to an independent group for which he created the new subfamily Sinolagomyinae Gureev 1960. Later some specimens of *S. kansuensis* were discovered in Oligocene faunas of China (Huang 1987; Wang and Qiu 2000). Also, new species were defined for Miocene faunas of the region; *Sinolagomys pachygnathus* Li et Qiu and *Sinolagomys ulungurensis* Tong (Li and Qiu 1980; Tong 1989). This demonstrated that the genus *Sinolagomys* survived the Oligocene–Miocene transition.

During the last decades comprehensive investigations of the joint Austrian–Mongolian Expeditions were conducted in the Valley of Lakes, Central Mongolia. Field work resulted in the collection of a great number of mammal fossils including lagomorphs in the region (Daxner-Höck et al. 2017, this issue), in particular an impressive number of ochotonid specimens of the genus *Sinolagomys* now stored in the NHMW (Vienna, Austria) and in the MPC/L (Ulaabaatar, Mongolia). Only fossils of the NHMW collection are considered in this study.

The present paper describes for the first time abundant fossil sinolagomyins collected from more than 50 localities of the Valley of Lakes, spanning in age the early late Oligocene to early Miocene. It is believed that the first archaic ochotonids represented by the genus *Sinolagomys* appeared by the latest early Oligocene. However, these are poorly preserved and uncommon, in contrast to other late Oligocene lagomorphs. Later, the genus flourished, with significant diversification and increasing abundance. Five Oligocene taxa are known in this region in addition to *S. tatalgolicus*. Among the ochotonid remains, there are several lower jaw fragments that differ from each other by structure of the valuable diagnostic tooth (p3) and can be recognised as different morphospecies, given the scarce materials. In the future with more material, these may be referred to independent taxa.

The genus continued to flourish through the late Oligocene–early Miocene demonstrating an exceptional transition of the Paleogene–Neogene boundary. The stratigraphic ranges of *Sinolagomys* species from Mongolia are shown in Table 1.

**Table 1:**
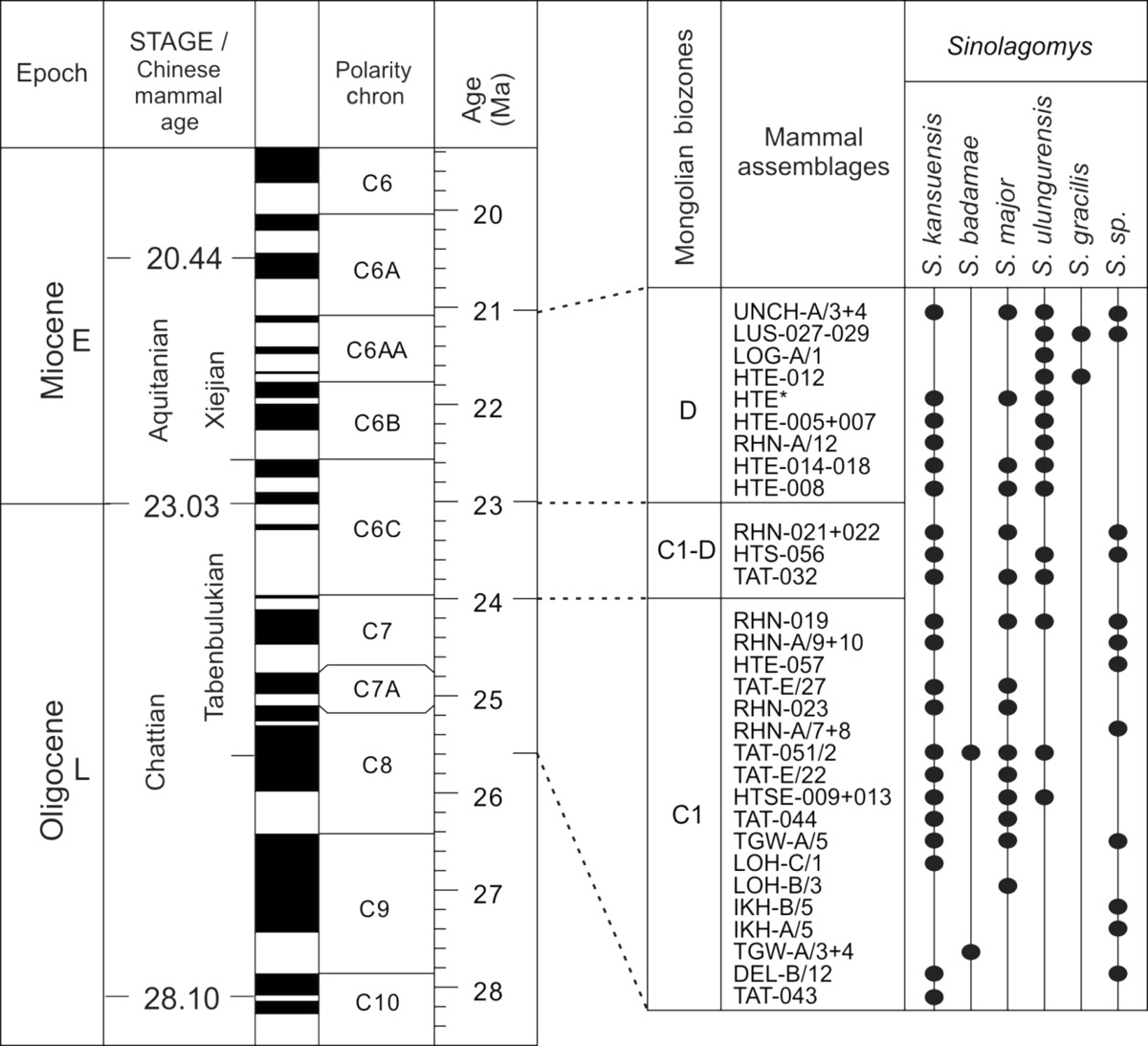
Stratigraphic chart including the geologic time scale (Gradstein et al. 2012), Mongolian biozones C1-D (Höck et al. 1999), the Europe MN/MP zones after Steininger (1999) and Luterbacher et al. (2004), the lower boundary of the Xiejian Chinese mammal age after Meng et al. (2013), the stratigraphic ranges of the genus *Sinolagomys* in Mongolia. Sporadic earlier occurrences of *S. major* and *S. kansuensis* are not considered in this chart

By then ochotonids (Ochotonidae) had dispersed over the huge territory of Asia from China and Mongolia in the east through Kazakhstan and beyond in the west (Li and Qiu 1980; Huang 1987; Tong 1989; Erbajeva 1988, 1994, 2007; Lopatin 1998; Wang and Qiu 2000; Meng et al. 2006, 2013; Bendukidze et al. 2009; Erbajeva and Daxner-Höck 2014; Erbajeva et al. 2016). Sinolagomyinae are a characteristic element of small mammalian assemblages of Central Mongolia (Valley of Lakes). They are a species-rich group playing an important role for palaeoenvironmental reconstruction in the region and they facilitate interregional correlations.

At the present time, the genus includes *S. kansuensis* Bohlin 1937 (type species), *S. major* Bohlin 1937, *S. gracilis* (Bohlin 1942), *S. tatalgolicus* Gureev 1960, *S. ulungurensis* Tong 1989, *S. pachygnathus* Li and Qiu 1980, and *Sinolagomys badamae* sp. nov.

## Material and methods

New material is stored in the collection of the Natural History Museum of Vienna, Vienna, Austria. A second rich lagomorph collection from the same fossil sites is housed in the Mongolian Paleontological Center, Academy of Sciences, Ulaanbaatar. Fossils were collected during eight field seasons since 1995 by teams of the Joint Austrian–Mongolian Projects (FWF: P-10505-GEO, P-15724-N06, and P-23061-N19) through screen washing and as surface finds on exposed deposits.

Type specimens and fossils of the genus *Sinolagomys* from the localities Shargaltein Tal, Taben Buluk, and Ulan Tatal were examined in the collections of the Institute of the Vertebrate Paleontology and Paleoanthropology, Chinese Academy of Sciences, Beijing, China. Type specimens of *S. tatalgolicus* from Tatal Gol and *Sinolagomys* sp. from Hsanda Gol were studied in the collections of the Paleontological Institute, Moscow, and American Museum of Natural History, New York, respectively.

The measurements (mm) were taken using MBS-10 microscope. The terminology of dental elements follows Lopez Martinez (1989) and in part Erbajeva (1988). The classification of Lagomorpha follows Gureev (1964), with the genus *Sinolagomys* placed in the subfamily Sinolagomyinae of the family Ochotonidae.

**Abbreviations**: NHMW—Museum of Natural History Vienna, Geological–Paleontological Department, Vienna, Austria

AMNH—American Museum of Natural History, New York, USA

IVPP—Institute of Vertebrate Paleontology and Paleo-anthropology, Chinese Academy of Sciences, China

MPC/L—Mongolian Paleontological Collection/Lagomorpha—collections of the Institute of Palaeontology and Geology, Mongolian Academy of Sciences, Ulaanbaatar, Mongolia

PIN—Paleontological Institute Russian Academy of Sciences, Moscow, Russia

**Teeth**: P, p—premolar; M, m—molar. Upper case letters indicate upper teeth; lower case for lower teeth. *L*—length and *W*—width of teeth, ant—anteroloph, hyp—hypostria, tr—trigonid, tal—talonid, *n*—number of specimen, *m*—mean, Min and Max—range of quantity, s—standard deviation

**Systematic Palaeontology**

Order Lagomorpha Brandt, 1855

Family Ochotonidae Thomas, 1897

Subfamily Sinolagomyinae Gureev, 1960

Genus *Sinolagomys* Bohlin, 1937

**Type locality**: Shargaltein Tal, Gansu (China), late Oligocene

Stratigraphic range: Late early Oligocene to early Miocene

**Diagnosis**: (after Bohlin 1937: 32; 1942: 95 with some corrections). Teeth small to medium-sized, moderately high crowned or hypsodont; no roots on upper teeth, rudimentary roots in lower teeth. P2: small; P3: paraflexus moderately deep; anteroloph short, not exceeding 1/3 of tooth width; internal hypostria short, with little cement; depth of hypostria on P4-M2 reaches half of tooth width or slightly more; M3: absent. Cross-section of p3 varies from rectangular to square; anterior margin flat or with vertical groove, shallow without cement varying to deep and filled with cement; antero-external fold filled with cement; trigonid of p4-m2 wider than talonid.

**Type species**: *Sinolagomys kansuensis* Bohlin, 1937; Holotype, P3-M2, Sh. 429. IVPP, Beijing, China

Referred species with type localities and geological age range:

*S. kansuensis* Bohlin, 1937; Shargaltein-Tal (China); late early Oligocene–early Miocene (type species)

*S. major* Bohlin, 1937; Shargaltein-Tal (China); late early Oligocene–early Miocene

*S. gracilis* (Bohlin 1942); Shargaltein-Tal (China); late Oligocene–early Miocene

*S. tatalgolicus* Gureev, 1960; Tatal-Gol (Mongolia); late Oligocene

*S. pachygnathus* Li and Qiu, 1980; Xiejia (China); early Miocene

*S. ulungurensis* Tong, 1989; Chibaerwoyi (China); late Oligocene–early Miocene

*S. badamae* sp. nov., Toglorhoi (Mongolia); late Oligocene

**Fig. 1:**
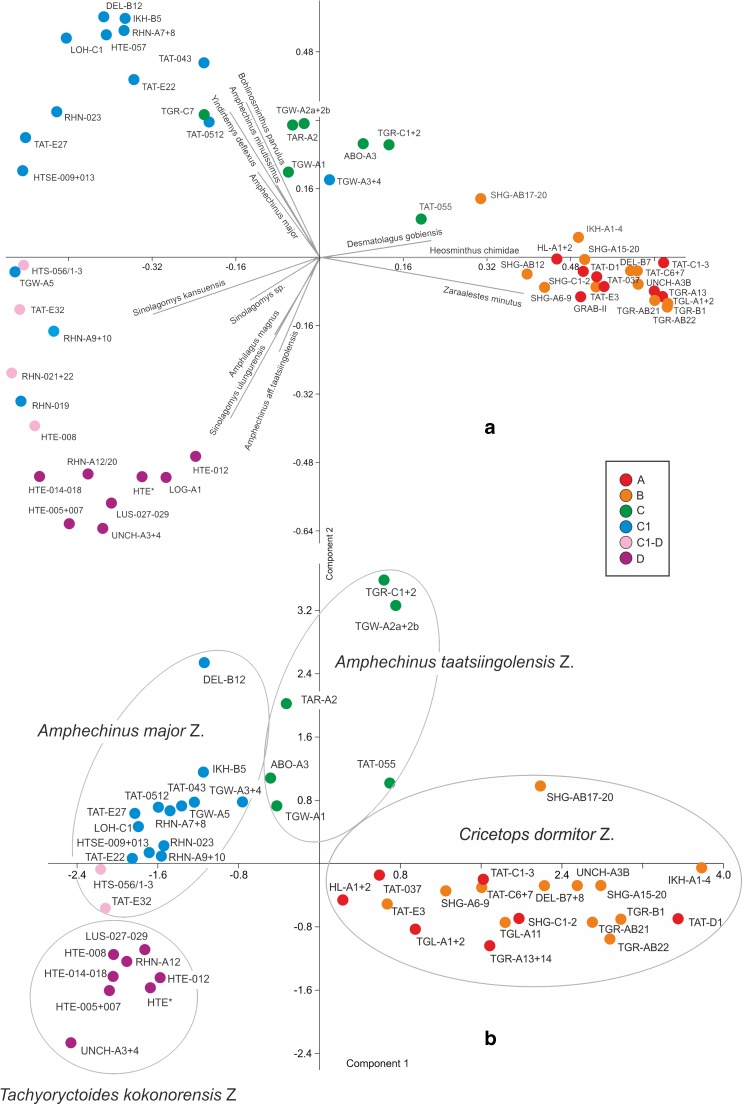
*Sinolagomys major* Bohlin, 1937 from the Valley of Lakes in Mongolia. **a** Right P3-M1 (NHMW 2013/0389/0088) from Hotuliin Teeg (HTE-008), early Miocene (biozone D). **b** Right P3-M1 (type, Sh. 830, IVPP, Beijing) from Shargaltein Tal, China, Late Oligocene

**Fig. 2:**
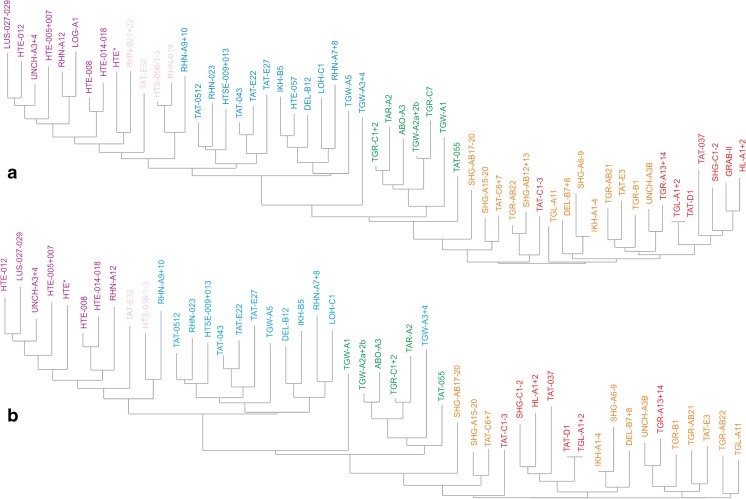
*Sinolagomys major* Bohlin, 1937 from the Valley of Lakes in Mongolia. **a** Right P3-M3 (NHMW 2013/0389/0089, from Hotuliin Teeg (HTE-008), early Miocene (biozone D). **b** Left p3 (NHMW 2013/0387/0008) from Huch Teeg (RHN-021), late Oligocene (biozone C1). **c** Left P4 (NHMW 2013/0386/0001) from Tatal Gol (TAT-52/2), late Oligocene (biozone C1). **d** Right M1 (NHMW 2013/0367/0005) from Huch Teeg (RHN-019), late Oligocene (biozone C1)

*Sinolagomys major* Bohlin, 1937 (Figures 1 and 2; Tables 2 and 3)

**Table 2:** Upper tooth measurements (in mm) of *Sinolagomys major* Bohlin, 1937

Nr	Specimens	*n*	*m*	Min	Max	*s*
1	P3-M3 L coronar	1	8			
2	P3-M2	5	7.25	7	7.5	0.18
3	P3-M1	6	5.58	5.4	5.8	0.157
4	P3-P4	5	3.63	3.4	3.75	0.140
5	P3	13	1.76	1.6	2	0.141
6	P3 W	13	2.88	2.5	3.1	0.168
7	P3 W ant	11	1.61	1.5	2	0.164
8	P4	22	1.79	1.65	2	0.104
9	P4 W	22	3.42	3	4	0.275
10	P4 W hyp	21	1.77	1.5	2.1	0.182
11	M1	11	1.74	1.5	2.15	0.161
12	M1 W	9	3.2	3	3.5	0.154
13	M1 W hyp	10	1.93	1.65	2.5	0.242
14	M2	8	1.58	1.45	1.65	0.065
15	M2 W	8	2.8	2.4	3.2	0.271
16	M2 W hyp	8	1.57	1.25	1.8	0.187
17	M3	1	0.55			
18	M3 W	1	1

**Table 3:** Lower tooth measurements (in mm) of *Sinolagomys major* Bohlin, 1937

Nr	Specimens	*n*	*m*	Min	Max	*s*
1	p4-m2	5	6.41	6.2	6.7	0.201
2	p3 L	6	1.53	1.45	1.6	0.061
3	p3 W	6	1.99	1.85	2.25	0.156
4	p4	20	2.2	1.75	2.55	0.234
5	p4 L tr	20	1.07	0.95	1.3	0.095
6	p4 W tr	20	2.29	1.5	2.8	0.336
7	p4 L tal	20	1.1	0.85	1.5	0.191
8	p4 W tal	19	1.8	1.55	2.15	0.191
9	m1	18	2.19	1.75	2.6	0.250
10	m1 L tr	18	1.11	0.9	1.35	0.132
11	m1 W tr	18	2.41	2	3	0.319
12	m1 L tal	18	1.04	0.8	1.25	0.143
13	m1 W tal	17	1.74	1.5	2	0.175
14	m2	15	2.37	1.6	2.8	0.339
15	m2 L tr	15	1.1	0.8	1.35	0.139
16	m2 W tr	14	2.46	1.8	3	0.348
17	m2 L tal	15	1.17	0.8	1.4	0.190
18	m2 W tal	14	1.79	1.2	2.1	0.255
19	m3 L	7	0.77	0.4	1	0.281
20	m3 W	7	0.97	0.45	1.25	0.328

1937 *Sinolagomys major*—Bohlin: 34–35, text-figs 58–61; Taf. 1. Fig. 18

1942 *Sinolagomys major*—Bohlin: 86–104, text-figs 24, 25*A*, ?*C*, 26A-C; 29; 31; 32 *A, B*

1987 *Sinolagomys major*—Huang: 272–275, text-fig. 12

1988 *Sinolagomys major*—Erbajeva: 50–51

2000 *Sinolagomys* cf. *S. major*—Wang and Qiu, pl. II, figs. 9–12

2014 *Sinolagomys*—Erbajeva and Daxner-Höck: 233–237, text-figs. 13–15

**Type locality**: Shargaltein Tal, Gansu (China), late Oligocene

**Type species**: *Sinolagomys major* Bohlin, 1937

**Holotype**: P3-M1, Sh. 830. IVPP, Beijing, China

**Stratigraphic range**: Late early Oligocene to early Miocene

**Emended diagnosis**: Large-sized; hypsodont teeth; rootless; upper cheek tooth crown slightly curved transversely; two thin plates (metasyle) follow from the occlusal surface of tooth towards root down the labial border of crown. On upper teeth (P4, M1, M2) depth of hypostria is variable. Lower p3 of rectangular shape, antero-external fold filled with cement; lower cheek teeth with trigonid wider than talonid.

**Referred material**: Fossils of *Sinolagomys major* are rather abundant. Studied materials are skull fragments, mandibles and isolated teeth sampled from the localities:

Early Oligocene (biozone B): Ikh Argalatyn Nuruu: IKH-A/1: M1 (NHMW 2016/0165/0001)

Late Oligocene (biozone C1):

Tatal Gol: TAT-044: 1 p3, 1 P4 (NHMW 2013/0105/0001-0002); TAT-052/1: 1 P4-M3, 1 P3 (NHMW 2013/0363/0001-0002); TAT-052/2: 1 P4 (NHMW 2013/0386/0001); TAT-E/22: 2 P4, 1 M2 (NHMW 2013/0278/0001-0003)

Toglorhoi: TGW-A/5: 1 M1 (NHMW 2016/0163/0001); TGS-A/O: 1 P4 (NHMW 2011/0194/0001)

Loh: LOH-B/3: 1 P4 (NHMW 2013/0364/0001)

Huch Teeg: RHN-A/7: 1 P4 (NHMW 2013/0366/0001); RHN-A/9: 1 P4 (NHMW 2016/0162/0001); RHN-019: 1 P3, 2 P4, 1 M1, 1 M2 (NHMW 2013/0367/0001-0005); HTSE-009:1 p4, 1 p3-m1 (NHMW 2013/0365/0001-0002); RHN-021: 2 P3-M2, 3 P3, 1 P4, 2 M1, 1 p3-m3, 1 m1-m3, 1 p3, 1 m1 (NHMW 2013/0387/0001-12); RHN-022: 1 P3-M2, 1 p3-m3 (NHMW 2013/0325/0001); HTSE-056: 1 P3 (NHMW 2013/0385/0001)

Early Miocene (biozone D):

Hotuliin Teeg: HTE-016-017: 1 m1, 1 m2 (NHMW 2013/0388/0001-0002); HTE-008: 1 P3-M3, 4 P3-M2, 1 P3-M1, 11 P3, 14 P4, 11 M1, 7 M2, 2 p3-m3, 4 p3-m2, 2 p3-p4, 1 p4-m3, 3 p4-m2, 2 p4-m1, 1 m1-m3, 2 m2-m3, 7 p3, 6 p4, 9 m1, 1 m2 (NHMW 2013/0389/0001-0089); HTE *: 1 P3-M2, 1 P4 (NHMW 2013/0195/0001-0002); HTE-009: 3 P4, 3 M1 (NHMW 2016/0164/0001-0006)

Huch Teeg: RHN-020: 3 P4 (NHMW 2013/0390/0001-0003)

Uncheltseg: UNCH-A/4: 1 P4 (NHMW 2014/0391/0001); UNCH-O: 1 P4 (NHMW 2011/0193/0002)

Description

The largest sinolagomyin, with hypsodont teeth; a marked ridge on the maxillary bone runs from the boundary of P3 and P4 alveoli towards zygomatic root; anterior and posterior to this ridge, there are depressions on the maxilla, relatively deep anterior and shallow posterior.

On upper teeth (P3, P4, M1, M2), two thin plates (metastyle) follow down from the occlusal surface towards root along height of tooth crown. It is safe to assume that such thin plates are rudiments of external roots that existed in the teeth of sinolagomyin ancestors.

Lingual borders of upper teeth (P4, M1, M2) slightly rounded; protocone larger than hypocone; enamel band is well developed across perimeter of tooth, but its thickness is variable.

**P2**: not known in our materials. However, Bohlin (1942, p. 92, T.b. 586a, fig. 27c) illustrates P2 with one anterior fold.

**P3**: oval-trapezoidal outline, tooth slightly longer on lingual part than labially, its anterior width significantly less than posterior width; paraflexus moderately deep; anteroloph extends to half of tooth width; internal hypostria short with little cement (Figs. 1a and 2a)

**P4**: in contrast to P3 tooth much wider anteriorly than posteriorly. No basic changes in P4 structure occur in *Sinolagomys major* from the late early Oligocene to early Miocene; all known specimens of P4 with the distinctive, characteristic significantly greater anterior width. Hypostria relatively short, extending across half of tooth width, with little cement; the type specimen from Shargaltein Tal (P3-M1, Sh. 830, fig. 58, Bohlin 1937, p. 34) is characteristic, with rather short hypostria (Figs. 1b and 2c).

**M1**: smaller than preceding tooth, anterior width of tooth slightly broader than, or equal to posterior width; enamel band well developed on the anterior and lingual margins of tooth; hypostria relatively deep, extends across half of tooth width or slightly more, filled with cement (Figs. 1a and 2d)

**M2**: small, anterior width of tooth exceeds posterior one; internal side of tooth inclined much towards postero-internal corner; hypostria deep, extends almost external to border of tooth (Fig. 2a)

**M3**: small, rounded, but usually lacking. The unique P3-M3 from the site HTE-008 (NHMW 2011/0389/0089) contains this tooth (Fig. 2a, Table 2).

Mandible relatively high, slightly robust (depth below p4 7.0–7.3 mm; width 5 mm), root of lower incisor extending to below the talonid of m2 forms marked convexity on both labial and lingual surface of mandible. Anterior foramen mentale located in front of p3 on the level of mid-height of mandible, the other two mental foramina are under p4. Enamel band well developed in all borders of lower teeth (p4-m2) except the anterior margin.

**p3**: rectangular shape with rounded margins; anterior border flat or with shallow depression with little or no cement; antero-external fold filled with little cement (Fig. 2b)

**p4-m2**: trigonids wider than talonids (Table 3) having oval shape, enamel band is thick on the posterior margin of conids.

**m3:** small oval conid, however in one specimen it is tiny (m2-m3, NHMW 2013/0194/0004). Similar small m3 was found in lower jaw of *Sinolagomys major* from Ulan Tatal (Huang 1987, fig. 12) and in one mandible of *Eurolagus* from La Grive, France.

**Discussion**: *S. major* from the Valley of Lakes resembles the type specimen from the locality Shargaltein Tal (Bohlin 1937, fig. 58; Sh. 830) in size and general structure of teeth, but differs by deeper hypostria of upper teeth; however, the ratio of the trigonid and talonid width in p4-m2 (Sh. 270, paratype) is similar.

A large ochotonid cited originally as *Lagomys* by Teilhard de Chardin (1926, fig. 14 A, p. 26), from the locality Saint-Jacques, Ordos, China, doubtlessly represents the genus *Sinolagomys*. Later, this specimen (P4 with relatively deep hypostria) was recognised by de Muizon (1977) as *Sinolagomys* cf. *major* Bohlin 1937.

*S. major* is known from the locality UlanTatal, China (Huang 1987). It is close to the population from the Valley of Lakes in size, the morphological structure of teeth, and lack of roots. This species is mentioned as well in the late Oligocene fauna of the northern Junggar basin, China (Meng et al. 2006).

*Ochotonolagus argyropuloi* Gureev 1960, large-sized ochotonid from Tatal Gol, Mongolia (Gureev 1960, Fig. 3a, b) resembles *Sinolagomys major* by the structure of the lower jaw general features of the occlusal surface of teeth (trigonids slightly wider than talonids), however it differs from the studied Mongolian form by retaining an oval shape m3 and by slightly smaller size. Lower cheek tooth length, p4-m3, is 6.7 mm in contrast to 7.3 mm in *S. major* from Loc. HTE-008. Lack of p3 in *O. argyropuloi* prevents definitive referral to *S. major*.

**Fig. 3:**
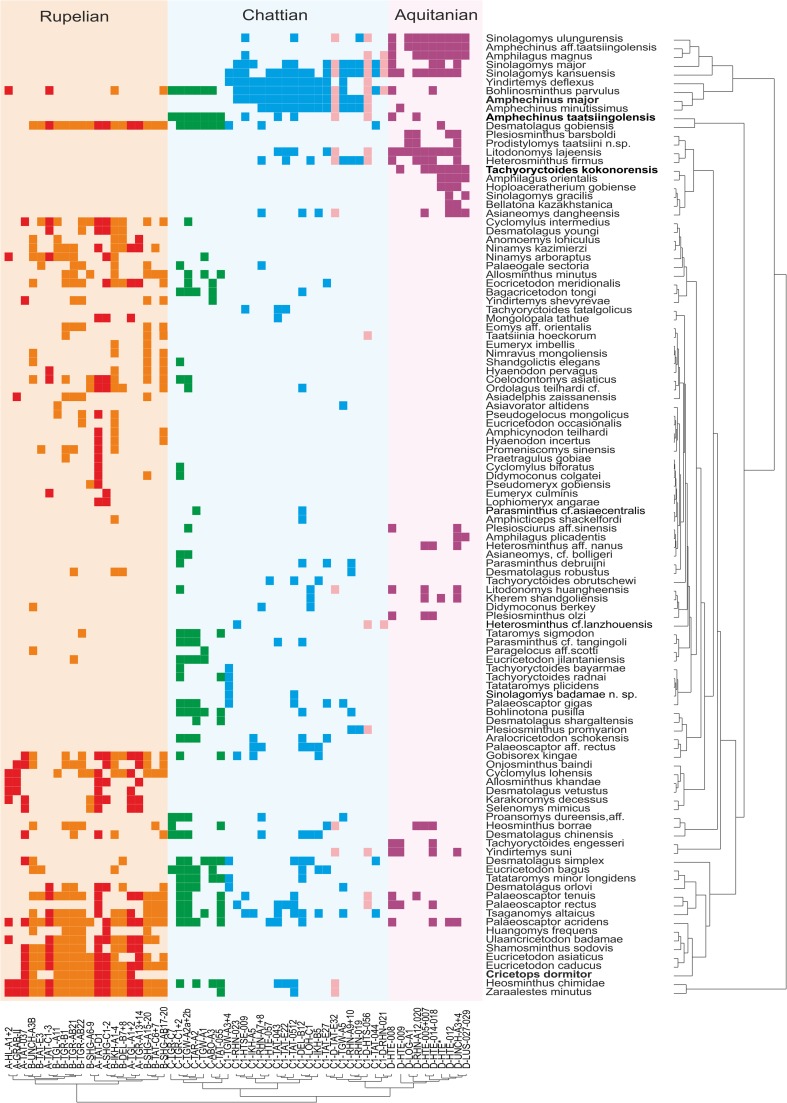
*Sinolagomys kansuensis* Bohlin, 1937 from the Valley of Lakes in Mongolia. **a** Left P3-M2 (NHMW 2013/0418/0011) from Huch Teeg (RHN-020), late Oligocene (biozone C1). **b** Right p3-m2 (NHMW 2013/0418/0002) from Huch Teeg (RHN-020), late Oligocene (biozone C1)

**Fig. 4:**
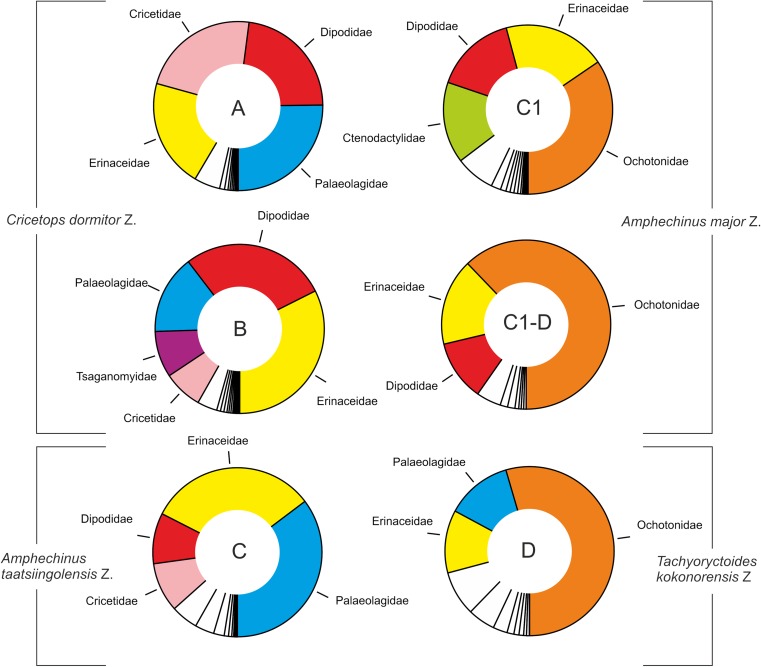
*Sinolagomys kansuensis* Bohlin, 1937 from the Valley of Lakes in Mongolia. **a** Right p3-m3 (NHMW 2013/0417/0003 from Hotuliin Teeg (HTE-0), early Miocene (biozone D) *Sinolagomys badamae* sp.nov. from the Valley of Lakes in Mongolia. **b** Right p3-m1 (NHMW 2011/0191/0001) from Toglorhoi (TGW-A), late Oligocene (biozone C–C1)

*Sinolagomys kansuensis* Bohlin, 1937 (Figures 3 and 4; Tables 4 and 5)

**Table 4:** Upper tooth measurements (in mm) of *Sinolagomys kansuensis* Bohlin, 1937

Nr	Specimens	*n*	*m*	Min	Max	*s*
1	P3	10	1.48	1.4	1.6	0.059
2	P3 W	10	2.43	2.25	2.8	0.174
3	P3 W ant	10	1.24	0.9	1.5	0.200
4	P4	5	1.48	1.4	1.55	0.057
5	P4 W	5	2.85	2.55	3.25	0.276
6	P4 W hyp	5	1.51	1.35	1.7	0.124
7	M1	9	1.44	1.3	1.6	0.111
8	M1 W	9	2.76	2.3	3.2	0.347
9	M1 W hyp	9	1.41	1	1.7	0.226
10	M2	2	1.43	1.35	1.5	0.106
11	M2 W	2	2.55	2.5	2.6	0.071
12	M2 W hyp	2	1.35	1.2	1.5	0.212

**Table 5:** Lower tooth measurements (in mm) of *Sinolagomys kansuensis* Bohlin, 1937

Nr	Specimens	*n*	*m*	Min	Max	*s*
1	p3-m3 L coronar	5	8.1	7.8	8.3	0.200
2	p3-m2	10	7.3	7.1	7.8	0.204
3	p3-m1	14	5.38	5	6	0.309
4	p3-p4	19	3.32	3	3.7	0.212
5	p4-m2	14	5.7	3.8	6.5	0.336
6	p3	37	1.26	1.1	1.4	0.057
7	p3 W	37	1.8	1.5	1.95	0.192
8	p4	35	1.93	1.6	2.2	0.157
9	p4 L tr	35	1.21	0.95	1.6	0.127
10	p4 W tr	35	2.12	1.75	2.5	0.193
11	p4 L tal	35	0.94	0.75	1.1	0.086
12	p4 W tal	35	1.59	1.25	1.8	0.131
13	m1	28	2.01	1.55	2.6	0.211
14	m1 L tr	28	1.03	0.75	1.25	0.099
15	m1 W tr	28	2.15	1.6	2.5	0.197
16	m1 L tal	28	0.96	0.75	1.3	0.105
17	m1 W tal	28	1.6	1.25	2	0.157
18	m2	18	2.07	1.75	2.5	0.168
19	m2 L tr	18	2.13	0.91	2.4	0.355
20	m2 W tr	18	2.14	1.75	2.5	0.203
21	m2 L tal	18	1.10	0.99	1.35	0.126
22	m2 W tal	18	1.62	1.45	1.8	0.088
23	m3 L	8	0.63	0.5	1	0.198
24	m3 W	8	0.98	0.8	1.2	0.151

1937 *Sinolagomys kansuensis* sp. nov.—Bohlin: 31–34, figs. 50–57

1942 *Sinolagomys kansuensis*—Bohlin: 96–99, pl. 1, figs. 18–31, text-figs. 24, 26

1987 *Sinolagomys kansuensis*—Huang: 272–274, text-fig. 11

1988 *Sinolagomys kansuensis*—Erbajeva: 49–50, text-fig. 10

2000 *Sinolagomys kansuensis*—Wang and Qiu: 262–259, pl. II, figs. 1–8

2009 *Sinolagomys kansuensis*—Bendukidze et al.: 349, text-figs. 3, 4

2014 *Sinolagomys kansuensis*—Erbajeva and Daxner-Höck:234–235, text-fig. 13

**Type locality**: Shargaltein Tal, Gansu (China), late Oligocene

**Type species**: *Sinolagomys kansuensis* Bohlin 1937

**Holotype**: P3-M2, Sh 429. IVPP, Beijing, China

**Stratigraphic range**: Late early Oligocene to early Miocene

**Emended diagnosis**: Medium-sized, high crowned teeth with reduced roots or rootless, tooth crown is relatively straight; on upper teeth (P4, M1, M2), the depth of hypostria is variable: on P4, it extends up to half of tooth width; in M1 and M2, it is deeper; p3 of quadrangular shape, anterior margin of tooth smooth or with shallow depression without cement, antero-external fold not deep but filled with cement; trigonid of p4-m2 is wider than talonid.

**Referred material:** the studied materials are represented by skull fragments, mandibles, and isolated teeth sampled from localities of Valley of Lakes, Mongolia:

Early Oligocene (biozone B): Hsanda Gol:

SHG-AB/17-20: 1 M1 (NHMW 2013/0358/0000)

Late Oligocene (biozone C): Taatsin Gol:

TGR-C/2: 1 p3-m2 (NHMW 2013/0419/0001)

Late Oligocene (biozone C1): Toglorhoi:

TGW-A/5, TGW-A/4-5: 1 P3-M1, 1 P3, 1 P4, 1 p3-m1 (NHMW 2013/0360/0001-0004); TGS-W: 1 P4, 1 p4, 1 m1 (NHMW 2013/0361/0001-0003)

Tatal Gol: TAT-E/22: 1 p4 (NHMW 2016/01598/0001); TAT-E/27: 1 P3, 1 P4, 5 M1, 1 M2, 1 m1 (NHMW 2014/0284/0001-0009); TAT-E/32: 1 P3, 1 p3 (NHMW 2014/0282/0001-0002);

Loh: LOH-C/1: 1 P3 2 P4, 1 M1, 1 m1, 1 m2 (NHMW 2013/0362/0001-0006)

Huch Teeg: RHN-A/7: 1 P3, 1 M2, 1 p4 (NHMW 2013/0243/0001-0003); RHN-A/9: 1 P3, 3 P4, 1 M1, 2 p3, 1 p4 (NHMW 2014/0242/0001-0008); RHN-019: 1 M2, 1 p3, 1 p4, 2 m1 (NHMW 2014/0269/0001-0005)

Hotuliin Teeg: HTSE-056/1-3: 4 P3, 1 P4, 1 M1, 1 p3 (NHMW 2014/0263/0001-0007)

Early Miocene (biozone D):

Hotuliin Teeg: HTE-005: 1 P3-M2, 3 P2, 8 P3, 1 P4, 2 M1, 2 M2, 1 p3-m2, 1 p3-p4, 1 p4-m2, 1 m1-m3, 1 m1-m2, 13 p3, 1 p4, 3 m1, 3 m2, 1 m3 (NHMW 2014/0285/0001-0043); HTE-008: 1 P2, 9 P3, 9 P4, 10 M1, 3 M2, 14 p4, 12 m1, 6 m2 (NHMW 2013/00156/0001-0064); HTE-014-015: 2 P2, 1 P3, 2 p4, 2 m1, 1 m2, 1 dp4 (NHMW 2014/0271/0001-0010); HTE-016-017: 4 P3, 3 P4, 3 M1, 2 M2, 3 p3, 1 p4, 3 M1, 2 M2, 1 M3, 1 dP2 (NHMW 2014/0261/0001-0023); HTE-012/7: 1 P3, 3 M1, 2 M2, 1 M2 (NHMW 2016/0158/0001-0007); HTE*: 1 p3-m3, 4 p3-m2, 2 p3-m1, 1 p3-p4, 1 p4-m3, 1 p4-m2, 1 p4-m1, 1 M1-m3 (NHMW 2013/0417/0001-0028)

Huch Teeg: RHN-020: 2 P3-M2, 1 P3-M1, 1 P3, 1 P4, 1 M1, 2 M2, 1 p3, 1 p4, 2 M1 (NHMW 2013/0418/0001-0013)

**Description:** Medium-sized ochotonid (Tables 4 and 5) with hypsodont teeth, most of the specimens rootless, but a few are rooted. Apparently the formation of roots occurs at an advanced ontogenetic stage.

**P3**: with moderately deep paraflexus; anteroloph short, extends to half or 1/3 of tooth width; small internal hypostria filled with little cement; protocone slightly larger than hypocone

In P4-M2, depth of hypostria is variable, filled with cement; in P4, hypostria does not exceed half of tooth width, but in M1 and M2, it is deeper. The enamel band well developed on the anterior and lingual margins of tooth (Fig. 3a).

Lower jaw is relatively robust; the incisor extends posteriorly along the ventral border of the mandible to the end of m1. The first foramen mentale is located slightly anteriorly to p3 and the second below m1.

**p3**: rectangular shape; anterior border of tooth is smooth or with shallow depression, without cement; antero-external fold filled with cement; the enamel band is missing on the external and posterior borders of tooth, whereas it is quite thick along anterior and lingual margins.

In lower teeth (p4-m2), trigonid is wider than oval shaped talonid (Figs. 3b and 4a).

**Discussion:** *Sinolagomys kansuensis* from the Valley of Lakes resembles the type species from the locality Shargaltein Tal, China, by the general structure of teeth, but differs significantly by lack of roots and by slightly larger size (Tables 4 and 5). Moreover, it differs by ratio of the trigonid and talonid width in p4-m2: in Mongolian specimens, the breadth of the trigonid is slightly larger than that of the talonid in contrast to the nominative species from China in which the trigonid is much wider than the talonid. The studied sample is similar to *S. kansuensis* from the locality UlanTatal, China, in size, in lack of roots and general morphological structure of teeth (Huang 1987, fig. 11).

Late Oligocene to early Miocene *S. kansuensis* dispersed westwards over the Northern Junggar Basin, China, and reached the Aral region in Kazakhstan. In the Junggar Basin, *S. kansuensis* is listed in the Teersihabahe and Suosuoquan Mammal assemblages (Meng et al. 2006, 2013). In Kazakhstan, the species is mentioned by Bendukidze et al. (2009) from localities Altyn Schokysu and Akotau. The Kazakh form resembles the Mongolian sample in similar structure of teeth and size, but differs in retaining rudimentary roots.

The comparative analysis of *S. kansuensis* from the Valley of Lakes and the type material from the Shargaltein Tal, China, allows us to hypothesize that by the end of the Oligocene or beginning of the Miocene, size increased slightly, the hypostria deepened, and roots were lost completely in this *Sinolagomys* lineage.

**Fig. 5:**
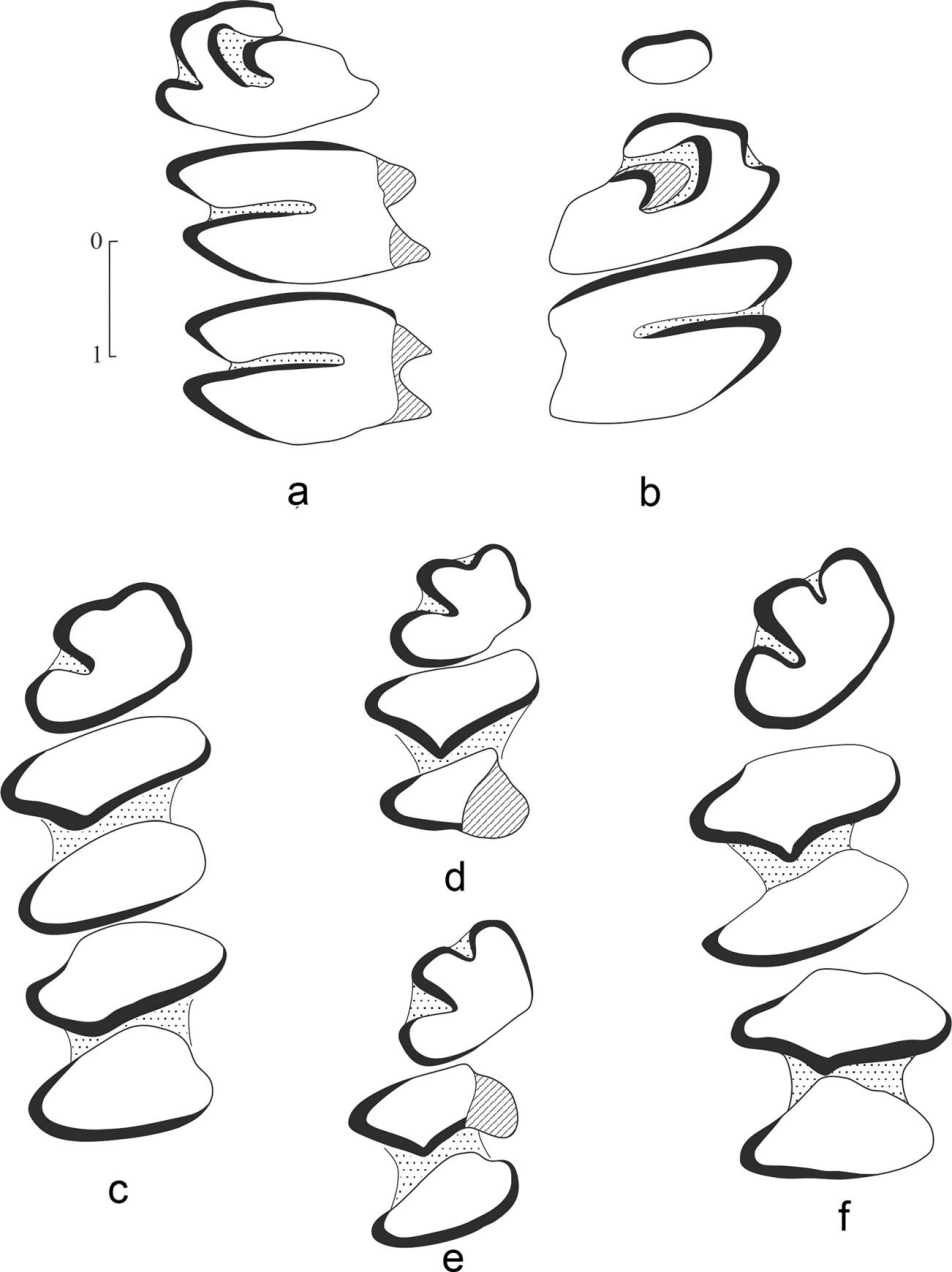
*Sinolagomys ulungurensis* Tong, 1989 from the Valley of Lakes in Mongolia. **a** Left P3-M1 (NHMW 2013/0376/0047) from Uncheltseg (UNCH-A/3), early Miocene (biozone D). **b** Right P2-P4 (NHMW 2013/0376/0001) from Uncheltseg (UNCH-A/3), early Miocene (biozone D). **c** Left p3-m1 (NHMW 2013/0376/0054) from Uncheltseg (UNCH-A/3), early Miocene (biozone D). **d** Left p3-p4 (NHMW 2013/0376/0003) from Uncheltseg (UNCH-A/3), early Miocene (biozone D). **e** Left p3-p4 (NHMW 2013/0376/0055) from Uncheltseg (UNCH-A/3), early Miocene (biozone D). **f** Left p3-m1 (NHMW 2014/0306/0001) from Del (DEL-WP 032), early Miocene (biozone D)

**Fig. 6:**
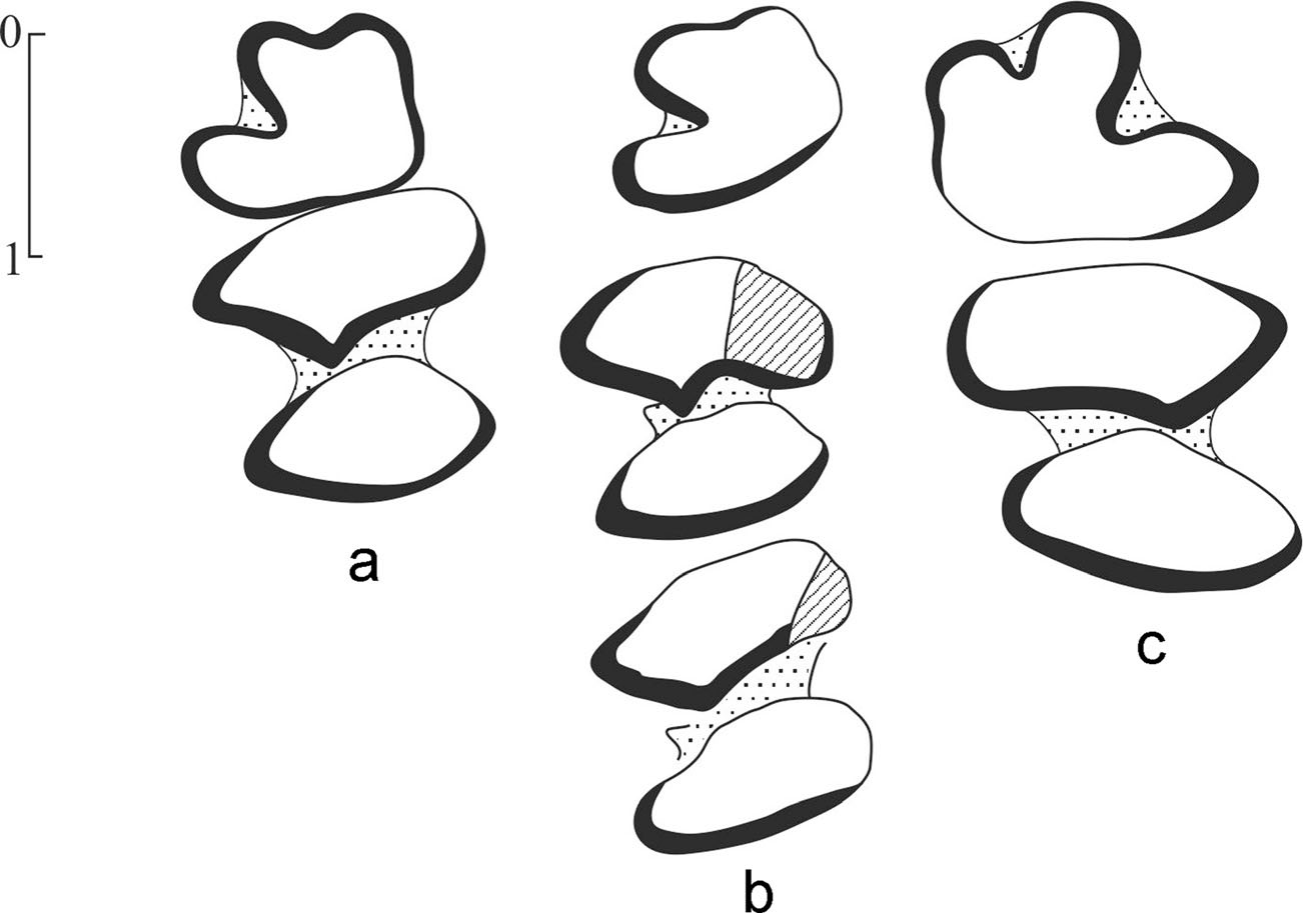
*Sinolagomys gracilis* (Bohlin, 1942) from the Valley of Lakes in Mongolia. **a** Left p3-p4 (MPC/L-0001) from Luuny Jas (LUS-029 = LUS-078), Mongolia, early Miocene (biozone D) *Sinolagomys gracilis* (Bohlin, 1942) from Shargaltein Tal, Gansu, China. **b** Left p3-m1 (type Sh.434, IVPP, Beijing) from Shargaltein Tal, China, Late Oligocene *Sinolagomys ulungurensis* Tong, 1989 from the Valley of Lakes in Mongolia. **c** Right p3-p4 (MPC/L-0002) from Luuny Jas (LUS-029 =LUS-078), Mongolia, early Miocene (biozone D)

*Sinolagomys ulungurensis* Tong, 1989 (Figures 5 and 6c; Tables 6 and 7)

**Table 6:** Upper tooth measurements (in mm) of *Sinolagomys ulungurensis* Tong, 1989

Nr	Specimens	*n*	*m*	Min	Max	*s*
1	P3-M2 L coronar	2	5.55	5.5	5.6	0.071
2	P3-M1	2	4.05	4	4.1	0.071
3	P3-P4	2	2.5	2.5	2.5	
4	P2	1	0.55			
5	P2 W	1	0.95			
6	P3	19	1.22	1	1.5	0.135
7	P3 W	19	2.19	1.75	2.6	0.223
8	P3 W ant	19	1.07	0.8	1.35	0.164
9	P4	11	1.35	1	1.75	0.222
10	P4 W	10	2.47	2.05	3.1	0.302
11	P4 W hyp	11	1.57	1.05	2.5	0.361
12	M1	12	1.28	1.05	1.55	0.144
13	M1 W	12	2.3	1.85	2.9	0.271
14	M1 W hyp	12	1.45	1.1	1.85	0.233
15	M2	7	1.06	0.95	1.3	0.117
16	M2 W	7	1.86	1.7	2.1	0.154
17	M2 W hyp	7	1.24	1	1.55	0.195

**Table 7:** Lower tooth measurements (in mm) of *Sinolagomys ulungurensis* Tong, 1989

Nr	Specimens	*n*	*m*	Min	Max	*s*
1	p3-m2 L coronar	3	5.58	5	6	0.520
2	p3-m1	5	3.83	3.45	4.3	0.390
3	p3-p4	6	2.43	2	3.2	0.427
4	p3	27	1.03	0.8	1.15	0.101
5	p3 W	27	1.38	0.9	1.6	0.189
6	p4	15	1.65	1.25	2	0.246
7	p4 L tr	15	0.85	0.65	1	0.131
8	p4 W tr	15	1.75	1.35	2.2	0.308
9	p4 L tal	15	0.79	0.6	1	0.113
10	p4 W tal	15	1.42	1.1	1.75	0.202
11	m1	26	1.64	1.3	2	0.199
12	m1 L tr	26	0.82	0.6	1.05	0.200
13	m1 W tr	26	1.64	0.75	2.25	0.297
14	m1 L tal	26	0.78	0.7	1.35	0.151
15	m1 W tal	26	1.38	1.05	1.6	0.154
16	m2	9	1.56	0.8	2.1	0.350
17	m2 L tr	9	0.94	0.7	1.7	0.313
18	m2 W tr	9	1.56	0.75	2.1	0.369
19	m2 L tal	9	0.83	0.7	1.3	0.187
20	m2 W tal	8	1.34	1.1	1.55	0.145
21	m3 L	1	0.55			
22	m3 W	1	0.85			

1989 *Sinolagomys ulungurensis—*Tong: 103–116; text-figs. 1–10

2014 *Sinolagomys ulungurensis*—Erbajeva and Daxner-Höck: 237, text-figs. 15/1–4

**Type locality**: North bank of the Ulungur River, Junggar basin, China, late Oligocene

**Type species:** *Sinolagomys ulungurensis* Tong, 1989

**Holotype:** P3-M2 (V 8264), IVPP, Beijing, China

**Stratigraphic range:** late Oligocene to early Miocene (biozones C1 to D)

**Emended diagnosis:** Small-sized; rootless, high crowned teeth; the depth of hypostria on upper teeth (P4, M1, M2) is variable, on P4 slightly exceeding half of tooth width, deeper in M1 and M2; p3 of rectangular shape, anterior margin of tooth is variable with shallow depression without cement or relatively deep depression filled with cement; antero-external fold with cement; trigonid of p4-m2 is slightly wider than talonid.

**Referred material:** occurrences of *Sinolagomys ulungurensis* in Valley of Lakes, Uvurkhangai Aimag

Late Oligocene (biozone C1):

Tatal Gol: TAT-E/32: 1 P3, 1 P4 1 M1, 1 M2, 2 p3, 1 p4, 1 m1, 1 m2 (NHMW 2014/0283/0001-0009); TAT-E/22: 1 p3, 1 p4 (NHMW 2014/0279/0001-0002)

Del: Del-B/12: 10 P3, 2 P4, 1 M1, 1 M2, 1 m3 (NHMW 2013/0368/0001-0015)

Huch Teeg: RHN-A/9: 1 P2, 1 P3, 1 P4, 1 p3 (NHMW 2014/0244/0001-0004); RHN-020: 1 P4, 1 p4 (NHMW 2013/0375/0001-0002)

Early Miocene (biozone D):

Luugar Khudag: LOG-A/1: 2 P3, 2 P4, 3 M1, 2 p3, 1 p4, 2 m2 (NHMW 2013/0373/0001-0012)

Luuny Yas: LUS-078: 15 P3, 13 P4, 5 M1, 4 M2, 9 p3, 21 m1, 3 m2, 1 m3 (NHMW 2014/0274/0001-0072); LUS-029: 4 P3, 3 P4, 6 M1, 2 M2, 1 p3-m2, 1 p3-m1, 3 m1, 1 m2, BM (NHMW 2014/0244/0001-0022)

Del: DEL-WP-032: 1 p3-m1 (NHMW 2014/0306/0001)

Hotuliin Teeg: HTE-surface: 1 P3, 1 M1, 2 m1-m2, 2 p3, 7 p4, 4 m1, 1 m2 (NHMW 2014/0275/0001-0019); HTE-005: 6 P3, 4 P4, 7 M1, 1 p3-p4, 1 p4-m1, 1 m1-m2 1 dP2 (NHMW 2013/0374/0001 0022); HTE-008: 4 P3, 3 p3 (NHMW 2014/0374/0023-0029); HTE-012: 3 P3, 1 P4, 4 M1, 2 M2, 2 M1, 1 m2 (NHMW 2014/0374/0030-0044); HTE 016–017: 1 P3 1 M2, 2 p3, 1 p4, 1 m1, 2 m2 (NHMW 2014/0374/0045-0052); HTE (TGN): 2 P3-M2, 1 p3-m3, 1 p3-m2, 1 p3-p4, 1 p4-m3, 1 m1-m2, 1 p3, 2 p4, 4 m1, 1 m2 (NHMW 2014/0374/0053-0068); HTE-012: 5 P3, 23 m1, 4 m2, 1 p3-m2, 1 p3-p4, 7 p3, 11 p4, 5 m1, 2 m2, BM (NHMW 2014/0374/0069-0128)

Huch Teeg: RHN-012: 1 P3, 1 P4, 1 M2, 2 p3, 1 p4 (NHMW 2013/0375/0001-0006

Unkheltseg: UNCH-A/3, UNCH-A/3 + 4: 2 P2-M2, 1 P2-P4, 3 P3-M2, 1 P3-M1, 1 P3-P4, 2 P4-M1, 1 dP2, 2 P2, 43 P3, 22 P4, 46 M1, 9 M2, 3 p3-m3, 3 p3-m2, 5 p3-m1, 8 p3-p4, 5 p4-m2, 2 p4-m1, 3 p4-m3, 4 m1-m3, 8 m1-m2, 2 m2-m3, 40 p3, 6 p4, 12 m1, 6 m2 (NHMW 2013/0376/0001-0212)

Description

Small-sized ochotonid (Tables 6 and 7), roots in teeth absent. P2 small, oval shape, with slight anterior groove without cement or lack of groove. As in *S. major* and *S. kansuensis*, the upper teeth (P3, P4, M1, M2) have two thin plates on the labial border (metastyle) running towards root from the occlusal surface.

Lingual borders of upper teeth (P4, M1, M2) slightly rounded; protocone and hypocone are almost equal in size; enamel band is well-developed across perimeter of tooth, but its thickness is variable—relatively thick in the anterior margin of upper teeth and posterior borders of lower teeth and thin respectively in posterior border of upper and anterior border of lower teeth.

**P2**: oval shape, with shallow or absent anterior groove, lacks cement (NHMW 2013/0376/0001 and NHMW 2013/0376/0121)

**P3**: with moderately deep paraflexus; anteroloph extends to mid-width of tooth; internal hypostria with little cement; protocone and hypocone of identical size; hypostria in upper teeth (P4-M2) filled with cement; depth of hypostria is variable: in P4 it reaches the middle of the tooth, and in M1 and M2 it is much deeper, may reach the external border of teeth (Fig. 5a, b).

Lower jaw relatively low, not robust; root of lower incisor extends to the talonid of m1; anterior foramen mentale located in front of p3 or below it at midlevel of mandible

**p3**: small, rectangular shape with rounded margins; anterior border with shallow depression without cement or with relatively deep and filled with cement. Antero-external fold with little cement. In p4-m2 trigonids wider than talonids, which are of oval shape (Figs. 5c–f and 6c)

**m3**: a small rounded, oval conid

**Discussion**: This small Mongolian ochotonid resembles the type series of *Sinolagomys ulungurensis* in general dental features and size of the teeth and undoubtedly belongs to this species. As in the type material, the Mongolian form is characterized by highly variable morphological structure of p3, in particular by the groove on the anterior margin of tooth which varies from shallow without cement to relatively deep and filled with cement.

*S. ulungurensis* is close to *S. gracilis* and *S. badamae* n. sp. in its small size, but it differs from them by p3 having a groove or depression on the anterior margin of tooth. It differs from the species *S. major*, *S. kansuensis*, *S. tatalgolicus*, and *S. pachygnathus* by its relatively smaller size, by its more complicated p3 with well-developed anterior grove or depression. Moreover, it differs from *S. kansuensis* by having rootless teeth.

*Sinolagomys gracilis* Bohlin, 1942 (Figures 6a, b, Table 8)

**Table 8:** Measurements of teeth (in mm) of *Sinolagomys gracilis* (Bohlin 1942)

	p3-m2	p3-m1	p3-p4	p3 L	p3 W	p4 L	p4 Wtr	p4 Wtal	P3 L	P3W
Type Sh 434	6.3	4.8	3. 15	1	1.35	1.5	1.5	1.35		
MPC/L-0001 (LUS-29)	4.75	3.35	2	0.8	1	1.35	1.25	1.15		
LUS-078									1.0;1.0	1.75;1.8
RHN-A/12									1.0	1.5

1937 *Sinolagomys minor* sp. nov.—Bohlin: 35, Taf. I, fig. 20, text-figs 62–65

1942 *Sinolagomys gracilis* n. sp.—Bohlin: Figs. 15n, ?23d, ?26f, w

**Type locality**: Shargaltein Tal, Gansu (China), late Oligocene

**Type species**: *Sinolagomys gracilis* n. sp., Bohlin 1942

**Holotype**: p3-m2 sin., Sh. 434 IVPP, Beijing, China

**Stratigraphic range**: late Oligocene to early Miocene

**Emended diagnosis**: Small-sized; teeth hypsodont, rootless; p3 rectangular in shape, anterior border of tooth smooth, with extremely shallow depression, close to straight; antero-external fold filled with cement, in p4-m2 width of talonid approaches that of the trigonid

**Referred material**: A fragment of left mandible with p3-m2 (MPC/L-0001)—collection of the Paleontological and Geological Institute, Mongolian Academy of Sciences, Ulaanbaatar, Mongolia—was collected by Demchig Badamgarav in 2012 from LUS-29

**Locality**: Luuny Jas (LUS-078 (=LUS-028/2011), P3 (right) NHMW 2016/ 0157/0001; P3 (right) NHMW 2016/0157/0002; Huch Teeg (RHN-A/12), P3 (right) NHMW 2016/ 0167/0001

**Age**: early Miocene (biozone D)

**Description**: Small-sized ochotonid, rootless, hypsodont teeth. P3 with relatively deep paraflexus; anteroloph moderately long, slightly exceeds half of tooth width; small internal hypostria with little cement; protocone slightly larger than hypocone. Metastyle small, follows from the occlusal surface downwards.

Lower jaw relatively gracile (width below p4 is 2.7 mm), lower incisor extends to below the trigonid of m2, forming a moderate tuberosity on the lingual side of mandible. Below the tuberosity is a well-marked depression, which gradually shallows towards the ascending ramus; lateral surface of mandible smooth. Anterior foramen mentale located below p3, slightly above the ventral border of the mandible, posterior foramen under boundary of p4 and m1.

Enamel band of p3 well-developed along perimeter of tooth except at the anterior margin (Fig. 6a, b)

Fossils of *S. gracilis* are not numerous in Mongolian faunas as is the pattern in China.

**Discussion**: A small ochotonid from Shargaltein Tal, Gansu, China, was described by Bohlin in 1937 as *Sinolagomys minor* (type Sh 96, P3-M1) on the basis of small size in comparison to *S. kansuensis* and *S. major*. Later, Bohlin (1942) revised all small ochotonid materials from Shargaltein Tal and Taben Buluk and he discovered that the type (Sh 96) cannot be distinguished from *S. kansuensis* by size. Given additional materials of small forms, he erected another new small-sized species *S. gracilis* based on another specimen (left p3-m2, Sh 434).

He wrote (Bohlin 1942, p. 100) “…the name *gracilis* was substituted for “minor” as the species to which this latter name (minor) was attached was based on doubtful material”. The fossils of *S. gracilis* in contrast to the other species (*S. kansuensis*, *S. major*), although scarce, are known from both localities Shargaltein Tal and Taben Buluk.

*S. gracilis* from the Valley of Lakes is close to the type material in the general structure of teeth and in having a similar ratio of the trigonid to talonid width; however, it differs from the type specimen by slightly smaller size (Fig. 6a, b; Table 8). This species differs from *S. kansuensis*, *S. major*, *S. tatalgolicus*, and *S. pachygnathus* by its much smaller size and by having talonid width practically equal to trigonid width in p4-m2. In other species, the trigonid of p4-m2 is much wider than the talonid.

*S. gracilis* differs from *S. badamae* sp. nov. and *S. ulungurensis* by slightly smaller size and from the latter by lacking a depression on the anterior border of p3. *S. badamae* sp. nov. differs from *S. gracilis* by having prominent, rounded anterior margin of p3. In addition, *S. kansuensis* differs from *S. gracilis* in its rooted teeth.

*Sinolagomys badamae* sp. nov. (Figure 4b, Table 9)

**Table 9:** Measurements of teeth (mm) of *Sinolagomys badamae* sp. nov

	p3-m1	p3-p4	p3	p4	m1	P3
*L*	4.0	2.5; 3.0	1.0; 1.18	1.5; 1.7; 1.7	1.5; 1.75	1.4
*W*			1.2; 1.4			2.3
Wtr				1.55; 1.75; 1.8	1.6; 1.8	
Wtal				1.15; 1.35; 1.4	1.1; 1.45	

**Derivatio nominis**: In honor of D. Badamgarav†, Mongolian co-leader of the research team and outstanding Mongolian geologist

**Type locality**: Toglorhoi, Uvurkhangai, Valley of Lakes, Mongolia; sample TGW-A* (asterisk indicates collection from surface along section TGW-A); red-brown silty clay of the Hsanda Gol Fm.; late Oligocene, Biozone C-C1

**Type species**: *Sinolagomys badamae* sp. nov.

**Holotype**: p3-m1 (right)—NHMW 2011/0191/0001, Natural History Museum, Vienna, Austria

**Age**: Late Oligocene

**Paratypes**: P3 (right) NHMW 2011/0191/0002; p3-p4 (right) NHMW 2011/0191/0003; p4 (left) NHMW 2011/0191/0004; m1 (right) NHMW 2011/0191/0005

**Diagnosis**: Small-sized rootless ochotonid with hypsodont teeth; anterior margin of p3 prominent (convex), rounded; trigonid in p4-m1 much wider than talonid.

**Differential diagnosis**: *Sinolagomys badamae* sp. nov. differs:

- From the largest species *S. major*, by much smaller size and prominent p3
- From *S. kansuensis* by smaller size and prominent p3, lack of roots in *S. badamae*
- From *S. pachygnathus* by prominent p3, smaller size and by the much wider trigonid than talonid in *S. badamae*
- From *S. gracilis* by slightly larger size, by greater breadth of trigonid relative to talonid (width of talonid in *S. gracilis* approaches that of the trigonid)
- From *S. ulungurensis* by lack of depression on the anterior margin of p3, by much wider trigonid than talonid (trigonid only slightly wider in *S. ulungurensis*)
- From *S tatalgolicus* by lack of deep depression on the anterior margin of p3 and smaller size

Description

Small-sized ochotonid (Table 9)

**P3**: rootless, oval-trapezoidal outline, its anterior width relatively less than posterior width; paraflexus moderately deep; anteroloph extends to 1/3 of tooth width; internal hypostria short, with little cement. Along external border of the tooth, thin plate follows from the occlusal surface towards root.

Mandible relatively high and robust (depth below p4 5.0–5.2 mm; width, 3.2–3.3 mm); the base of lower jaw is wide; the lower incisor extends to the talonid of m1 forming marked tuberosity on both lateral and medial surfaces of mandible. Anterior foramen mentale located anterior to p3 at mid-level of mandible. The thickness of enamel band in lower teeth (p3-m1) varies: it is well developed along the perimeter of teeth, except for the anterior margin of trigonid and lingual part of talonid.

**p3**: rectangular shape with rounded borders; anterior margin of tooth prominent, lingual and posterior margins are practically straight; anteroconid elongated; enamel band is well developed on all borders of tooth; antero-external fold of tooth is relatively shallow, with little cement.

**p4-m1**: trigonid significantly wider than talonid; in the holotype, both labial and lingual edges of trigonid in p4 and m1 are relatively sharpened to acute angles, but in the paratype, they are slightly rounded; in all specimens, the posterior margin of the trigonid has a short sharp projection and the structure of the talonid is the same; talonid is oval or egg-shaped, with rounded lingual and sharp labial edges, enamel band well developed along posterior border of talonids.

**Discussion**: *Sinolagomys badamae* sp. nov. differs from all other species of the genus by the morphological structures of teeth and size as noted above. The species is characterized by advanced features such as lack of roots and the peculiar, prominent anterior portion of p3; however, some archaic characters are retained as well. Archaic features are much wider trigonid than talonid, lower incisor extending far posteriorly (to the talonid of m1), and mandible relatively robust despite the small size.

## Conclusion

Fossils of *Sinolagomys* are rather well-preserved across the Oligocene to Miocene informal biozones C1–D of the Valley of Lakes in Central Mongolia. First very rare occurrences are evidenced in biozone B (two specimens) and in biozone C (one specimen), but the main distribution of the genus was during the biozones C1, C1-D, and D. The comprehensive analysis of the entire collection shows that several Oligocene species were distributed from the northern part of China to Central Mongolia and Eastern Kazakhstan. The diversity and abundance of Sinolagomyinae is highest in Biozone C1, and they appear to be an endemic taxon of Central Asia.

The Neogene record of *Sinolagomys* in Central Asia continues in the early Miocene with the appearance of *S. ulungurensis* alongside the Oligocene species *S. major* and *S. kansuensis*.

No significant morphological changes in tooth structure of *S. major* and *S. kansuensis* from the Valley of Lakes sites are observed during the early late Oligocene and Miocene. Possibly, these species were rather conservative forms that persisted on landscapes under environments that did not change greatly.

The fossil history of the Ochotonidae offers clues to the palaeoclimate of the mid-Cenozoic world, especially for the palaeoecology of eastern Asia. The diversity patterns observed among fossil representatives indicate the changing conditions of dominant climatic patterns. Heightened fossil diversity is slowly emerging and indicates an increasing component of open habitat in a mosaic of grasslands and woodlands during the middle Cenozoic in Asia. This led to the development of the main ochotonid adaptive type. Inhabiting open landscapes and taking grass as nourishment, sinolagomyins are distinguished by increasing hypsodonty of the teeth at the early stage of their evolutionary development. Well-developed roots were lost as crown height increased, and root closure occurred late in ontogeny, yielding “rudimentary roots” (closed pulp cavities at the base of teeth). Later, “rudimentary roots” were lost completely as the teeth became fully hypsodont with open pulp cavities.

Erbajeva et al. (2015) summarized much of the known record of Neogene and late Paleogene ochotonids, especially Asian records. There are additional important records of early Ochotoninae, such as early Miocene *Alloptox* (*Mizuhoptox*) from Japan (Tomida 2012). Apparently, the Neogene was the time of diversification and increasing abundance of advanced ochotonids. Oligocene Sinolagomyinae, rather than Ochotoninae, characterized late Paleogene assemblages of Asia. Now it is clearer that Sinolagomyinae were actually rather abundant in the late Oligocene and early Miocene of Asia. A hypothetical scenario for ochotonid evolution may be proposed.

A long, sustained period of global cooling characterized the late Eocene, leading to climatic oscillations during the Oligocene Epoch (“Zoogeography of Paleogene Asia” 1974; Berggren and Prothero 1992; Zachos et al. 2001). Until the late Oligocene, early ochotonids were uncommon elements in terrestrial communities. With global cooling and growing seasonality of precipitation, habitats changed, becoming more open, and primitive ochotonids of the subfamily Sinolagomyinae flourished. Locally they became common, as in the Valley of Lakes, Mongolia (Tab. 1). The genus *Sinolagomys* is the earliest species-rich form of the subfamily, and it flourished during the late Oligocene and early Miocene. We hypothesize *Sinolagomys* as a characteristic element of small mammal assemblages of eastern Asia in which subtropical forests had been replaced by landscapes with open woodlands and xerophytic vegetation.
